# Antimicrobial Activities of Sesquiterpene-Rich Essential Oils of Two Medicinal Plants, *Lannea egregia* and *Emilia sonchifolia*, from Nigeria

**DOI:** 10.3390/plants10030488

**Published:** 2021-03-05

**Authors:** Akintayo L. Ogundajo, Tolulope Ewekeye, Olubunmi J. Sharaibi, Moses S. Owolabi, Noura S. Dosoky, William N. Setzer

**Affiliations:** 1 Department of Chemistry, Natural Products Research Unit, Faculty of Science, Lagos State University, Badagry- Expressway, P.M.B. 0001 LASU Post Office, Ojo, Lagos 100268, Nigeria; ogundajotayo@yahoo.com; 2 Department of Botany, Faculty of Science, Lagos State University, Badagry- Expressway, P.M.B. 0001 LASU Post Office, Ojo, Lagos 100268, Nigeria; tolulope.ewekeye@lasu.edu.ng (T.E.); bjlawal2002@yahoo.com (O.J.S.); 3 Aromatic Plant Research Center, 230 N 1200 E, Suite 100, Lehi, UT 84043, USA; ndosoky@aromaticplant.org; 4 Department of Chemistry, University of Alabama in Huntsville, Huntsville, AL 35803, USA

**Keywords:** α-panasinsen, γ-himachalene, (*E*)-caryophyllene, α-copaene, selena-4,11-diene, antibacterial, antifungal

## Abstract

*Lannea egregia* (Anacardiaceae) and *Emilia sonchifolia* (Asteraceae) are plants used in traditional medicine in southwestern Nigeria. The essential oils from the leaves of *L. egregia* and *E. sonchifolia* were obtained by hydrodistillation and analyzed by gas chromatography–mass spectrometry. Both essential oils were dominated by sesquiterpenoids. The major components in *L. egregia* leaf essential oil were α-panasinsen (34.90%), (*E*)-caryophyllene (12.25%), α-copaene (11.39%), and selina-4,11-diene (9.29%), while *E. sonchifolia* essential oil was rich in γ-himachalene (25.16%), (*E*)-caryophyllene (15.72%), and γ-gurjunene (8.58%). The essential oils were screened for antimicrobial activity against a panel of bacteria and fungi and displayed minimum inhibitory concentrations ranging from 156 μg/mL to 625 μg/mL. Based on these results, either *L. egregia* or *E. sonchifolia* essential oil may be recommended for exploration as complementary antibacterial or antifungal agents.

## 1. Introduction

Medicinal plants are widely used in treatment of diseases, and this has encouraged researchers to investigate plants that are of pharmacological value with potential therapeutic application in the management of human health [1]. Many ethnomedicinal plants have been investigated and reported to possess antiviral [2], anticancer [3], antiprotozoal [4], antibacterial [5], antifungal [6], anti-inflammatory [7], antioxidant [8] and other biocidal activities [9,10,11]; hence, their usefulness in folk medicine for treatment of various diseases has given credence to the application of the ethnopharmacological approaches for drug discovery.

The genus *Lannea* is in the family Anacardiaceae, which consists of nearly 800 species in 82 genera. There are around 40 *Lannea* species distributed across the savanna region of the West African tropics from Guinea through Ghana to Nigeria [12,13]. Several important members of *Lannea* species include *L. kerstingii, L. welwitschii, L. schimperii, L. egregia, L. acida, L. microcarpa,* and *L. fruticosa* [14]. *Lannea egregia* Engl. and K. Krause, locally called “ekudan” in Yoruba in Nigeria, “sambituliga” in Ivory Coast, and “tiuko” in Guinea [15], is a tropical woody perennial plant about 13 m in height with alternate leaves growing in the savanna region and shares the same local name as *L. barteri* (Oliv.) Engl., in Ivory Coast, Benin, and Guinea [16].

Ethnomedicinally, *L. egregia* has seen traditional use in treatment of various ailments in humans. The roots and bark are used externally for ulcers, sores, and leprosy [12]. The plant decoction is taken as a remedy for diarrhea, edema, epilepsy, rheumatism, insanity, paralysis, and gastric pains [17,18], as well as to improve the hemoglobin level and as part of vermifuge medicine [16]. The macerated roots have been used to treat wounds [19]. Traditionally, leaves of *L. egregia*, boiled with fermented corn water, were used for treatment of hemorrhoids [19] and to manage cancer [20]. The leaf, stem bark, and root extracts of *L. egregia* from Olokemeji Forest, Nigeria, were shown to have antibacterial activity against *Staphylococcus aureus*, *Bacillus subtilis*, *Pseudomonas aeruginosa*, and *Escherichia coli* using a zone-of-inhibition assay, but showed only weak activity with minimum inhibitory concentrations (MIC) ranging from 6.3 to 25 mg/mL [21]. In this work, a phytochemical screening was carried out, but individual components were not identified.

*Emilia sonchifolia* (L.) DC. (Asteraceae) is a bushy annual herb distributed mainly in Asian countries, but naturalized throughout the tropics [13]. It has been traditionally used as an important medicinal plant in most tropical and subtropical countries [22], including in the South-South region of Akwa Ibom State, Nigeria [23,24]. The plant has been used to treat diarrhea, night blindness, sore throat, chest pain, liver disease, eye inflammation, stomach tumor, rashes, measles, earache, inflammation, convulsions, fever, muscular aches, and asthma [24,25,26]. There have been several studies reported in the literature on the biological activities and phytochemical screening of extracts of *E. sonchifolia* (Table 1). The plant extracts have shown anti-inflammatory, antioxidant, cytotoxic, analgesic, wound-healing, antimetastatic, immunomodulatory, and antiangiogenic activities [27].

We report herein our investigation into the collection of *L. egregia* leaf essential oil and the essential oil from the leaves of *E. sonchifolia* from southwest Nigeria, the analysis of the essential oil compositions, and antimicrobial screening of the essential oils. This investigation is part of our ongoing research aimed at the characterization of the bioactivity and the compositions of the essential oils from Nigerian medicinal plants for potential exploitation in pharmaceutical applications.

## 2. Results and Discussion

### 2.1. Essential Oil Compositions

#### 2.1.1. Lannea egregia

**Figure 1:**
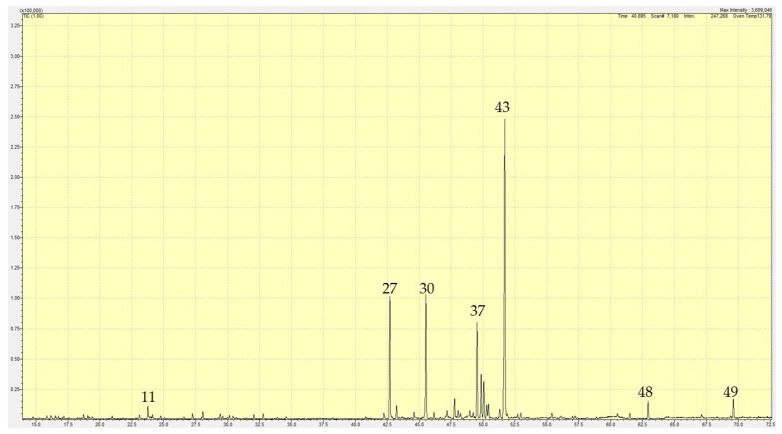
Gas chromatogram of *Lannea egregia* leaf essential oil. Major compounds are indicated by Sr. Nos. from Table 2.

Hydrodistillation of the leaves of *L. egregia* collected from Agbegi-Odofin Village, Ikire, Osun State, Nigeria, yielded a pale-yellow essential oil with an average yield of 0.68 ± 0.2% on a weight-to-weight basis. The essential oil was analyzed by gas chromatography—mass spectrometry (GC-MS) (Table 2, Figure 1). The essential oil showed monoterpene hydrocarbons (1.53%), oxygenated monoterpenoids (2.86%), sesquiterpene hydrocarbons (86.43%), oxygenated sesquiterpenoids (1.15%), and non-terpenoids (6.50%). The predominant sesquiterpene hydrocarbons include α-panasinsen (34.90%), (*E*)-caryophyllene (12.25%), α-copaene (11.39%), and selina-4,11-diene (9.29%). The major oxygenated monoterpenoid was linalool (1.12%).

As far as we are aware, there have been no published reports on essential oils from *Lannea* species, so essential oil compositional comparisons at the genus level are not possible. Both α-copaene and (*E*)-caryophyllene are common essential oil components, including the Anacardiaceae (see, for example [35,36]). Selin-4,11-diene, on the other hand, is relatively uncommon in the family, but has been observed in *Sclerocarya birrea* leaf essential oil [37] and *Haematostaphis barteri* leaf essential oil [38]. Likewise, α-panasinsen is a rare volatile component in the Anacardiaceae, but detected as an aroma component of *Mangifera indica* cv. Alphonso [39] and *Sclerocarya birrea* subsp. *caffra* [40] fruits.

#### 2.1.2. Emilia sonchifolia

**Figure 2:**
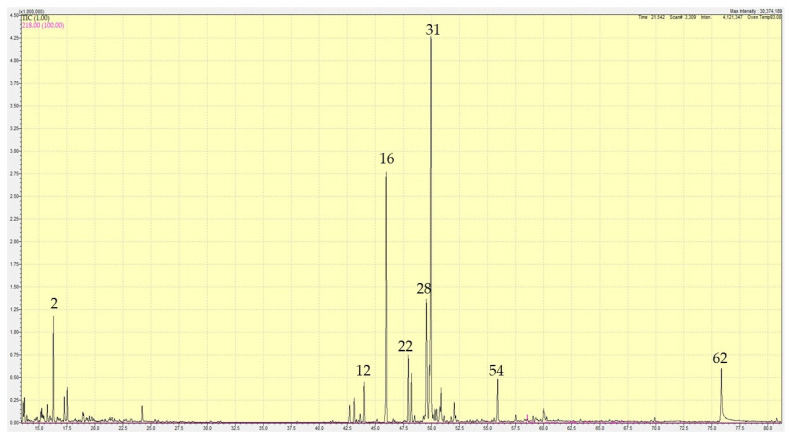
Gas chromatogram of *Emilia sonchifolia* leaf essential oil. Major compounds are indicated by Sr. Nos. from Table 3.

Hydrodistillation of the leaves of *E. sonchifolia* yielded a pale-yellow essential oil (0.46%). A total of 62 constituents, 97.60% of *E. sonchifolia* volatile oil, were identified by GC-MS. The volatile oil composition is displayed in Table 3 and visualized in Figure 2. The leaf oil was dominated by sesquiterpenoids: γ-himachalene (25.16%), (*E*)-caryophyllene (15.72%), γ-gurjunene (8.58%), (*E*)-β-farnesene (3.96%), germacrene D (3.53%), and caryophyllene oxide (3.05%), in addition to the fatty acid palmitic acid (5.24%), and the monoterpene β-pinene (4.87%).

The essential oil of the aerial parts of *E. sonchifolia* from Belagavi, Karnataka, India, has been reported [41]. The essential oil from India was also dominated by sesquiterpene hydrocarbons (67.6%), but with a remarkably different composition. The major components in the essential oil from India were γ-muurolene (32.1%) and (*E*)-caryophyllene (22.7%). γ-Muurolene was not observed in the essential oil from Nigeria, while γ-himachalene, γ-gurjunene, and germacrene D were not reported in the essential oil from India. Both caryophyllene oxide and palmitic acid were found in the essential oil from India (1.1% and 1.2%, respectively). Apparently, the geographical separation of these two samples has a profound effect on the phytochemistry.

Both *L. egregia* and *E. sonchifolia* essential oils were dominated by sesquiterpene hydrocarbons, with (*E*)-caryophyllene abundant in both oils. α-Copaene, abundant in *L. egregia* essential oil, was found to be only 1.5% in *E. sonchifolia* oil. Selina-4,11-diene and α-panasinsen were major components in *L. egregia* essential oil but were not detected in the essential oil of *E. sonchifolia*. Likewise, γ-himachalene, abundant in *E. sonchifolia* essential oil, was not detected in the essential oil of *L. egregia*.

### 2.2. Antimicrobial Activity

The leaf essential oils of *L. egregia* and *E. sonchifolia* were screened for antibacterial and antifungal activity against a panel of microorganisms (Table 4). It has been suggested that essential oils having MIC values < 100 μg/mL show very strong activity, those with MIC of 101–500 μg/mL show strong activity, 500 μg/mL < MIC < 1000 μg/mL are moderately active, and above 1000 μg/mL are inactive [42,43]. Thus, the essential oils in this study can be considered strongly active. It is not readily apparent which essential oil components are responsible for the activities; most sesquiterpenes have not been individually screened for antimicrobial activity. However, three of the major components, β-pinene, linalool, and (*E*)-caryophyllene, were also screened in this work and these compounds showed activities similar to the essential oils themselves. The observed antimicrobial activities are consistent with some of the ethnobotanical uses of these two plants, and based on these results, either *L. egregia* or *E. sonchifolia* essential oil may be recommended for exploration as antibacterial or antifungal agents.

## 3. Materials and Methods

### 3.1. Plant Materials

Leaves of *Lannea egregia* and *Emila sonchifolia* were collected directly from source plants in two locations in southwestern states in Nigeria in the month of August, 2019. *Lannea egregia* was collected from Agbegi-Odofin Village, Ikire (Osun State, 7°22′20.68″ N, 4°11′14.60″ E), and *E. sonchifolia* was obtained from the campus of Lagos State University, Ojo (Lagos State, 6°28′1.20″ N, 3°10′58.80″ E). Botanical identification of the two plants was done by Mr. S. A. Odewo at the Herbarium, Forest Research Institute of Nigeria (FRIN), Jericho, Ibadan, Nigeria, where their voucher specimens (Voucher Numbers FHI 112544 and FHI 112546, respectively) have been deposited. The leaves *L. egregia* and *E. sonchifolia* were manually removed, chopped, air-dried in the laboratory for 7–10 days, pulverized using an electric blender, and stored in polyethene containers until ready for use.

### 3.2. Isolation of Essential Oils

A sample (450 g each) of *L. egregia* leaves and *E. sonchifolia* leaves was subjected to hydrodistillation thrice in an all-glass Clevenger-type apparatus. Each sample of *L. egregia* and *E. sonchifolia*, respectively, was mixed with water in a ratio of 2:6. The mixture was hydrodistilled for 3–4 h with constant stirring until no additional oil was observed to be distilled. For each plant species, the essential oils were combined, dried over anhydrous sodium sulfate to eliminate traces of water, and stored in a sealed vial under refrigeration (4 °C) prior to analysis.

### 3.3. Gas Chromatography–Mass Spectrometry

The leaf essential oils of *L. egregia* and *E. sonchifolia* were analyzed using gas chromatography–mass spectrometry (GC-MS) as previously described by us [38]: Shimadzu GCMS-QP2010 Ultra, ZB-5 ms GC column, GC oven temperature 50 °C–260 °C (2 °C/min), 1-μL injection of 5% solution of each essential oil dissolved in CH_2_Cl_2_ (split mode, 30:1). Each essential oil sample was injected three times. Retention indices (RI) were calculated in comparison with a homologous series of *n*-alkanes. Compounds were identified by comparison of the MS fragmentation and retention indices with those in the databases [44,45,46,47] and with matching factors >90%. Quantification was done by external standard method. Calibration curves of representative compounds from each class were drawn and used for quantification.

### 3.4. Antibacterial and Antifungal Screening

The essential oils were screened for antimicrobial activity against a panel of bacteria (*Bacillus cereus* (ATCC No. 14579), *Staphylococcus aureus* (ATCC No. 29213), and *Staphylococcus epidermidis* (ATCC No. 12228), *Streptococcus pyogenes* (ATCC No. 19615), and fungi (*Aspergillus fumigatus* (ATCC No. 96918), *Aspergillus niger* (ATCC No. 16888), *Cryptococcus neoformans* (ATCC No. 32045), *Microsporum canis* (ATCC No. 11621), *Microsporum gypseum* (ATCC No. 24102), *Trichophyton mentagrophytes* (ATCC No. 18748), *Trichophyton rubrum* (ATCC No. 28188), and *Candida albicans* (ATCC No. 18804)) using the microbroth dilution technique [48,49] as previously reported by us [38]. Serial dilutions of the essential oils (2500, 1250, 625, 312.5, 156.3, 78.1, 39.1, and 19.5 μg/mL) in appropriate media (cation–adjusted Mueller Hinton broth for bacteria and yeast-nitrogen base growth medium for fungi) were carried out in 96-well microtiter plates. Microorganisms (1.5 × 10^8^ CFU/mL for bacteria and 7.5 × 10^7^ CFU/mL for fungi) were added to the 96-well plates, which were incubated for 24 h at 37 °C for bacteria and 35 °C for fungi. Minimum inhibitory concentrations (MIC) were determined to be the lowest concentrations without turbidity. Gentamicin (Sigma-Aldrich, St. Louis, MO) was the positive antibacterial control, amphotericin B (Sigma-Aldrich, St. Louis, MO) was the positive antifungal control, and dimethylsulfoxide (DMSO) was used as the negative control (50 μL DMSO diluted in 50 μL broth medium, and then serially diluted as above). (–)-β-Pinene, (±)-linalool, (*E*)-caryophyllene, and caryophyllene oxide (Sigma-Aldrich, St. Louis, MO) were also individually screened for activity.

## 4. Conclusions

The essential oils of *Lannea egregia* and *Emilia sonchifolia*, medicinal plants collected from southwestern Nigeria, were found to be rich in sesquiterpenoids. Both essential oils exhibited antibacterial and antifungal activities that are consistent with traditional uses of the plants. While sesquiterpene hydrocarbons were the predominant chemical class in both essential oils, it is not apparent which individual components may be responsible for the antimicrobial activity. It is likely, however, that synergistic effects are also responsible for the activities of the components. Nevertheless, the essential oils may be recommended for further exploration as complementary antimicrobial agents.

## Figures and Tables

**Table 1:** Biological activities of *Emilia sonchifolia* extracts.

*Emilia sonchifolia* Extract (Geographical Source)	Phytochemicals Identified	Biological Activity	Ref.
Methanol plant extract (Kerala, India)	None identified	In vitro cytotoxicity (L-929 murine lung fibroblast, IC_50_ = 15 μg/mL)	[28]
Aqueous leaf extract (Nsukka, Nigeria)	None identified	Anti-inflammatory (mouse paw edema assay, ED_50_ = 780 mg/kg)	[23]
Ethanol plant extract (Kerala, India)	None identified	Inhibition of perchlorate oxidative stress (rat model)	[29]
Methanol leaf extract (Ibiono, Nigeria)	None identified	Analgesic (acetic acid writing, formalin hind paw, and hot plate assays, mouse model)	[25]
CH_3_OH/CH_2_Cl_2_ (1:1) extract of aerial parts (Nsukka, Nigeria)	Quercetrin, chlorophyll, caffeic acid derivative	Anti-inflammatory (inhibition of pro-inflammatory cytokines, mouse model)	[30]
Ethanol extract of aerial parts (Liuzhou, China)	Emiline (pyrrolidine alkaloid)	Neuroprotective (in vitro PC12 cells)	[31]
Aqueous HCl (0.5 N) plant extract (Taiwan)	Pyrrolizidine alkaloids: senecionine, seneciphylline, integerrimine, senkirkine, otosenine, neosenkirkine, petasitenine, acetylsenkirkine, desacetyldoronine, acetylpetasitenine, and doronine	None carried out, but pyrrolizidine alkaloids known to be hepatotoxic.	[32]
Aqueous plant extract (Kerala, India)	None identified	Wound-healing activity (rat model)	[33]
Ethanol leaf extract (Abraka, Nigeria)	None identified	Antifungal activity (*Curvularia lunatus*, MIC = 72 mg/mL)	[34]
Methanol leaf extract (Uyo, Nigeria)	None identified	Antioxidant (FRAP and DPPH assays)	[26]

IC_50_ = Median inhibitory concentration. ED_50_ = Median effective dose. FRAP = Ferric ion Reducing Antioxidant Power. DPPH = 2,2-diphenyl-1-picrylhydrazyl.

**Table 2:** The chemical constituents of *Lannea egregia* leaf essential oil.

Sr. No.	RT	RI(calc)	RI(db)	Compound	Ave %	St Dev
1	15.838	977	978	β-Pinene	0.21	0.06
2	16.152	983	986	6-Methylhept-5-en-2-one	0.38	0.04
3	16.494	989	991	2-Pentylfuran	0.25	0.02
4	18.725	1024	1025	*p*-Cymene	0.34	0.03
5	19.044	1028	1030	Limonene	0.24	0.02
6	19.149	1030	1031	β-Phellandrene	0.11	0.01
7	20.961	1057	1058	γ-Terpinene	0.22	0.01
8	21.811	1070	1069	*cis*-Linalool oxide	0.12	0.02
9	22.81	1085	1086	Terpinolene	0.08	0.02
10	23.099	1089	1091	*p*-Cymenene	0.32	0.04
11	23.752	1099	1099	Linalool	1.12	0.01
12	23.979	1102	1104	Hotrienol	0.13	0.02
13	24.116	1104	1104	Nonanal	0.30	0.02
14	24.759	1113	1112	(*E*)-2,4-Dimethylhepta-2,4-dienal	0.26	0.04
15	26.577	1139	1139	(*Z*)-3-Ethylidene-1-methyl-1,4-cycloheptadiene	0.22	0.02
16	27.252	1149	---	Unidentified	0.44	0.02
17	28.064	1160	1169	*p*-Dimethoxybenzene	0.54	0.06
18	29.26	1177	1172	Lavandulol	0.12	0.02
19	29.416	1180	1180	Terpinen-4-ol	0.44	0.05
20	30.132	1190	1192	Methyl salicylate	0.30	0.00
21	30.415	1194	1195	α-Terpineol	0.26	0.05
22	32.05	1218	1219	β-Cyclocitral	0.42	0.03
23	32.783	1228	---	Unidentified	0.47	0.05
24	34.591	1255	1257	Carvenone	0.25	0.01
25	40.81	1347	1349	α-Cubebene	0.20	0.02
26	42.245	1369	1367	Cyclosativene	0.61	0.06
27	42.704	1376	1375	α-Copaene	11.39	0.22
28	43.231	1384	1382	β-Bourbonene	1.25	0.06
29	44.584	1404	---	Unidentified	0.61	0.05
30	45.524	1419	1417	(*E*)-Caryophyllene	12.25	0.10
31	46.156	1429	1430	β-Copaene	0.57	0.02
32	47.186	1446	1447	Geranyl acetone	0.62	0.06
33	47.774	1455	1454	α-Humulene	1.85	0.03
34	48.052	1460	1457	*allo*-Aromadendrene	0.65	0.04
35	48.978	1474	1478	γ-Muurolene	0.57	0.03
36	49.235	1478	1479	α-Amorphene	0.41	0.06
37	49.533	1483	1476	Selina-4,11-diene	9.29	0.03
38	49.853	1488	1492	β-Selinene	4.26	0.04
39	50.065	1492	1492	Valencene	3.86	0.09
40	50.295	1495	1497	α-Selinene	1.24	0.08
41	50.425	1497	1497	α-Muurolene	1.35	0.06
42	51.313	1512	1512	γ-Cadinene	0.88	0.05
43	51.704	1519	1521	α-Panasinsen	34.90	0.25
44	52.716	1536	1538	α-Cadinene	0.37	0.05
45	52.96	1540	1541	α-Calacorene	0.54	0.02
46	55.41	1581	1577	Caryophyllene oxide	0.60	0.05
47	61.503	1688	1694	Acorenone B	0.55	0.06
48	62.926	1714	1715	Pentadecanal	1.82	0.12
49	69.609	1840	1841	Phytone	1.81	0.06
				Total identified	98.48	

RT = retention time (min); RI(calc) = retention index determined with respect to a homologous series of *n*-alkanes on a ZB-5 ms column; RI(db) = retention indices from the databases. Monoterpene hydrocarbons (Sr. Nos. 1, 4–7, 9, 10), 1.53%; oxygenated monoterpenoids (Sr. Nos. 8, 11, 12, 18, 19, 21, 22, 24), 2.86%; sesquiterpene hydrocarbons (Sr. Nos. 25–28, 30, 31, 33–45), 86.43%; oxygenated sesquiterpenoids (Sr. Nos. 46, 47), 1.15%; others (Sr. Nos. 2, 3, 13–15, 17, 20, 32, 48, 49), 6.50%. Sr. No. 16 MS(EI): 152(7%), 137(37%), 119(27%), 109(100%), 93(16%), 91(28%), 81(33%), 79(26%), 77(21%), 67(91%), 55(23%), 43(71%), 41(30%). Sr. No. 23 MS(EI): 151(2%), 136(26%), 121(72%), 108(43%), 93(100%), 91(32%), 79(32%), 77(25%), 43(39%), 41(23%). Sr. No. 29 MS(EI): 225(2%), 210(44%), 195(100%), 182(12%), 167(66%), 152(20%), 137(18%), 125(29%), 82(15%), 70(18%), 69(13%), 56(16%), 55(20%), 54(26%), 43(19%), 42(17%), 41(21%).

**Table 3:** Chemical composition of *Emilia sonchifolia* leaf essential oil.

Sr. No.	RT	RI(calc)	RI(db)	Compound	Ave %	St Dev
1	13.703	924	921	Tricyclene	1.08	0.16
2	16.285	971	974	β-Pinene	4.87	0.04
3	19.510	1027	1024	Limonene	0.25	0.01
4	41.005	1343	1345	7-*epi*-Silphiperfol-5-ene	0.07	0.00
5	41.195	1346	1345	α-Cubebene	0.11	0.00
6	41.445	1350	1350	α-Longipinene	0.04	0.00
7	42.700	1369	1369	Cyclosativene	1.19	0.02
8	43.105	1375	1374	α-Copaene	1.46	0.01
9	43.305	1378	1374	Isoledene	0.18	0.00
10	43.640	1384	1387	β-Bourbonene	0.50	0.01
11	43.865	1387	1385	α-Bourbonene	0.08	0.00
12	43.985	1389	1389	β-Elemene	2.38	0.05
13	44.170	1392	1390	Sativene	0.04	0.01
14	44.895	1403	1398	Cyperene	0.06	0.00
15	45.140	1407	1409	α-Gurjunene	0.20	0.01
16	45.960	1420	1417	(*E*)-Caryophyllene	15.72	0.35
17	46.585	1430	1430	β-Copaene	0.23	0.01
18	46.725	1432	1432	*trans*-α-Bergamotene	0.10	0.01
19	47.485	1444	1447	Isogermacrene D	0.05	0.00
20	47.580	1446	1445	Myltayl-4(12)-ene	0.14	0.00
21	47.700	1448	1444	6,9-Guaiadiene	0.09	0.00
22	47.925	1452	1454	(*E*)-β-Farnesene	3.96	0.03
23	48.205	1456	1452	α-Humulene	2.97	0.03
24	48.490	1461	1464	9-*epi*-(*E*)-Caryophyllene	0.42	0.05
25	48.620	1463	1465	*cis*-Muurola-4(14),5-diene	0.04	0.02
26	49.285	1474	1476	Selina-4,11-diene	0.49	0.03
27	49.410	1476	1479	γ-Muurolene	0.41	0.01
28	49.540	1478	1475	γ-Gurjunene	8.58	0.36
29	49.605	1479	1483	α-Amorphene	2.81	0.57
30	49.820	1482	1484	Germacrene D	3.53	0.10
31	49.945	1484	1481	γ-Himachalene	25.16	0.78
32	50.040	1486	1487	Aristolochene	0.61	0.13
33	50.140	1488	1492	δ-Selinene	0.52	0.03
34	50.305	1490	1489	β-Selinene	0.81	0.02
35	50.455	1493	1496	Valencene	0.97	0.03
36	50.745	1498	1498	α-Selinene	1.19	0.03
37	50.855	1499	1500	α-Muurolene	2.11	0.04
38	50.965	1501	1505	α-Cuprenene	0.16	0.03
39	51.120	1504	1505	(*E*,*E*)-α-Farnesene	0.38	0.05
40	51.375	1508	1505	β-Bisabolene	0.13	0.04
41	51.590	1512	1509	Tridecanal	0.06	0.01
42	51.755	1514	1513	γ-Cadinene	0.35	0.01
43	51.925	1517	1514	Cubebol	0.08	0.01
44	52.030	1519	1522	δ-Cadinene	1.29	0.04
45	52.140	1521	1520	7-epi-α-Selinene	0.41	0.03
46	52.255	1523	1521	*trans*-Calamenene	0.05	0.01
47	52.345	1524	1521	β-Sesquiphellandrene	0.16	0.02
48	53.165	1538	1537	α-Cadinene	0.10	0.01
49	53.445	1543	1544	α-Calacorene	0.18	0.01
50	53.680	1544	1545	*trans*-Cadinene ether	0.11	0.00
51	53.845	1547	1548	α-Elemol	0.04	0.00
52	54.680	1561	1564	β-Calacorene	0.05	0.01
53	55.580	1576	1577	Spathulenol	0.32	0.02
54	55.890	1581	1582	Caryophyllene oxide	3.05	0.03
55	57.510	1609	1608	Humulene epoxide II	0.39	0.01
56	59.300	1641	1638	τ-Cadinol	0.21	0.01
57	59.430	1643	1640	τ-Muurolol	0.13	0.00
59	60.055	1654	1652	Himachalol	0.49	0.05
59	59.980	1653	---	Unidentified	0.86	0.05
60	60.255	1658	1658	*neo*-Intermedeol	0.32	0.00
61	69.900	1839	1841	Phytone	0.27	0.01
62	75.845	1960	1959	Palmitic acid	5.24	1.25
63	80.780	2066	2071	Dibenzyl disulfide	0.23	0.00
				Total identified	97.60	

RT = retention time (min); RI(calc) = retention index determined with respect to a homologous series of *n*-alkanes on a ZB-5 ms column; RI(db) = retention indices from the databases. Monoterpene hydrocarbons (Sr. Nos. 1–3), 6.20%; sesquiterpene hydrocarbons (Sr. Nos. 4–40, 42, 44–49, 52), 80.47%; oxygenated sesquiterpenoids (Sr. Nos. 43, 50, 51, 53–57, 59, 60), 5.13%; others (Sr. Nos. 41, 61, 62, 63), 5.80%. Sr. No. 59 MS(EI): 206(4%), 191(5%), 173(3%), 163(7%), 149(9%), 136(14%), 135(13%), 124(22%), 123(32%), 121(19%), 109(100%), 95(39%), 93(28%), 81(33%), 79(28%), 69(24%), 67(46%), 55(30%), 53(18%), 43(18%), 41(47%).

**Table 4:** Antibacterial and antifungal activities (MIC, μg/mL) of *Lannea egregia* and *Emilia sonchifolia* essential oils from southwest Nigeria.

Organism	*Lannea egregia* EO	*Emilia sonchifolia* EO	(–)-β-Pinene	(±)-Linalool	(*E*)-Caryophyllene	Caryophyllene Oxide	Positive Control ^a^
Bacteria							
*Bacillus cereus*	312.5	625	312.5	312.5	312.5	312.5	1.22
*Staphylococcus aureus*	312.5	1250	256.3	312.5	312.5	78.1	0.61
*Staphylococcus epidermidis*	312.5	156.3	312.5	312.5	312.5	312.5	<19.5
*Streptococcus pyogenes*	625	312.5	625	312.5	312.5	625	<19.5
Molds							
*Aspergillus fumigatus*	156.3	156.3	156.3	156.3	156.3	156.3	<19.5
*Aspergillus niger*	156.3	156.3	78.1	1250	1250	156.3	1.56
*Cryptococcus neoformans*	312.5	625	312.5	312.5	312.5	312.5	0.78
*Microsporum canis*	312.5	312.5	312.5	312.5	312.5	312.5	<19.5
*Microsporum gypseum*	312.5	312.5	312.5	312.5	312.5	156.3	<19.5
*Trichophyton mentagrophytes*	156.3	312.5	156.3	625	625	156.3	<19.5
*Trichophyton rubrum*	312.5	312.5	312.5	312.5	312.5	312.5	<19.5
Yeast							
*Candida albicans*	156.3	312.5	156.3	156.3	156.3	312.5	1.56

MIC = minimum inhibitory concentration (μg/mL). ^a^ Gentamicin was the positive control for bacteria, amphotericin B was the positive control for fungi.

## Data Availability

All data are contained within the article.

## Abbreviations

ATCC: American Type Culture Collection; CFU, colony forming units; DPPH, 2,2-diphenyl-1-picrylhydrazyl; ED_50_, median effective dose; EI, electron impact; FRAP, ferric reducing antioxidant power; GC, gas chromatography; IC_50_, median inhibitory concentration; MIC, minimum inhibitory concentration; MS, mass spectrometry; RI, retention index/retention indices; RT, retention time.
